# Different association of GDF15 and FGF21 with glycemic status and aging in the context of obesity

**DOI:** 10.1007/s11357-025-01830-3

**Published:** 2025-08-19

**Authors:** Laura Salmón-Gómez, Victoria Catalán, Beatriz Ramírez, Maite Aguas-Ayesa, Amaia Rodríguez, Sara Becerril, Jorge Baixauli, Sonsoles Gutiérrez-Medina, Carmen Mugueta, Inmaculada Colina, Carolina M. Perdomo, Camilo Silva, Javier Escalada, Gema Frühbeck, Javier Gómez-Ambrosi

**Affiliations:** 1 Metabolic Research Laboratory, Clínica Universidad de Navarra, Pamplona, Spain; 2 Centro de Investigación Biomédica en Red-Fisiopatología de La Obesidad y Nutrición (CIBEROBN), Instituto de Salud Carlos III, Pamplona, Spain; 3 Obesity and Adipobiology Group, Instituto de Investigación Sanitaria de Navarra (IdiSNA), Pamplona, Spain; 4 Institute of Nutrition & Health, University of Navarra, Pamplona, Spain; 5 Department of Endocrinology & Nutrition, Clínica Universidad de Navarra, Pamplona, Spain; 6 Department of Surgery, Clínica Universidad de Navarra, Pamplona, Spain; 7 Department of Endocrinology & Nutrition, Clínica Universidad de Navarra, Madrid, Spain; 8 Department of Biochemistry, Clínica Universidad de Navarra, Pamplona, Spain; 9 Department of Internal Medicine, Clínica Universidad de Navarra, Pamplona, Spain

**Keywords:** GDF15, FGF21, Type 2 diabetes, Obesity, Aging, FGF21/adiponectin ratio

## Abstract

Growth differentiation factor 15 (GDF15) and fibroblast growth factor 21 (FGF21) are stress-induced cytokines increased in age-related and metabolic disorders. The aim of the present study was to analyze the association between circulating concentrations of GDF15 and FGF21 in obesity and type 2 diabetes (T2D) in the context of aging. Serum samples from 405 participants (33 with normal weight and normoglycemia (NG), 156 with obesity and NG, 157 with obesity and impaired glucose tolerance (IGT), and 59 with obesity and T2D) aged 47 ± 13 years, were recruited to study the serum GDF15 and FGF21 concentrations and their relationship with obesity, T2D, and aging. Adipokines (leptin and adiponectin) and other cardiometabolic risk factors were also measured. The circulating concentrations of GDF15 and FGF21 were significantly increased with obesity and further increased with IGT and T2D. Both GDF15 and FGF21 showed positive associations with glucose (*r* = 0.25, *P* < 0.001 and *r* = 0.40, *P* < 0.001, respectively) and insulin (*r* = 0.16, *P* < 0.01 and *r* = 0.27, *P* < 0.001, respectively) levels. Similarly, GDF15 and FGF21 were positively correlated with age (*r* = 0.55, *P* < 0.001 and *r* = 0.15, *P* < 0.01, respectively). Interestingly, the FGF21/adiponectin and GDF15/adiponectin ratios emerged as good biomarkers for the presence of T2D (AUC = 0.807 and 0.779, respectively). Our findings support that obesity, T2D, and aging increase the concentrations of both GDF15 and FGF21. Furthermore, GDF15 concentrations are more associated with aging while FGF21 levels are more related to the metabolic status. Finally, we propose the FGF21/adiponectin ratio as a novel biomarker for the detection of the presence of T2D.

**Supplementary Information:**

The online version contains supplementary material available at 10.1007/s11357-025-01830-3.

## Introduction

The rising prevalence of obesity over the past few decades has established this condition as a serious threat to public health, and it is currently considered a new pandemic [1, 2]. Obesity is a multifactorial disease associated with an excess of adipose tissue (AT) and characterized by chronic low-grade inflammation [3, 4]. Obesity increases the risk of developing other diseases such as metabolic syndrome, type 2 diabetes (T2D), cardiovascular diseases, obstructive sleep apnea, or certain types of cancer, which can increase the mortality risk. Among them, T2D is the most closely linked to obesity [5, 6]. One of the main benefits of treating obesity is the improvement or remission of obesity-associated comorbidities that accompany weight loss [7]. Nevertheless, despite advances in treatment with bariatric surgery and incretin-based pharmacological therapies, long-term maintenance of weight loss and metabolic control remains difficult to achieve for many people with obesity [8]. Therefore, prompt detection of the possible development of obesity-associated complications is essential to prevent their progression. Hence, new effective treatments and biomarkers for early diagnosis still need to be developed.

In this context, growth differentiation factor 15 (GDF15) and fibroblast growth factor 21 (FGF21) are currently in the spotlight. Both are cytokines whose levels increase in humans under stress conditions [9]. GDF15 belongs to the transforming growth factor $\beta$ (TGF-$\beta$) superfamily and regulates energy balance by suppressing food intake [10, 11] and enhancing energy expenditure in muscle during caloric restriction [12]. To mediate metabolic regulation, GDF15 interacts with its receptor, the glial cell-derived neurotrophic factor (GDNF) family receptor $\alpha$-like (GFRAL) [13–16]. Although GFRAL expression appeared to be limited to the brainstem, it has also been observed in some peripheral tissues, such as the testes and AT [16, 17]. Unlike its receptor, GDF15 is expressed in several tissues under physiological conditions, including liver, intestine, kidneys, and placenta, and its levels are elevated in various pathologies, such as obesity and T2D and other age-related disorders [10, 13, 18]. In this regard, an increase in GDF15 concentrations with age, independently of health status, has been demonstrated [19, 20], while sustained high levels of GDF15 have been associated with weight loss in human diseases [21]. However, due to the increase in its levels, obesity has been suggested as a possible state of resistance to GDF15 [22]. Additionally, a positive association has been observed between baseline GDF15 circulating levels and the risk of T2D development, suggesting that GDF15 may be useful for identifying people at risk of developing T2D [23].

FGF21 belongs to the fibroblast growth factor (FGF) family, with key functions in cellular and metabolic homeostasis [24]. FGF21 regulates energy homeostasis primarily by enhancing energy expenditure [25]. To mediate these effects, FGF21 binds to fibroblast growth factor receptor (FGFR) and to $\beta$-Klotho, which is a co-receptor protein, to activate FGFR signaling activity [26]. Although FGF21 is expressed in several tissues such as AT, pancreas, or skeletal muscle, circulating FGF21 levels mainly derive from the liver [25] and its levels are increased by different stressors such as cold, exercise, mitochondrial stress, and nutritional stress including obesity, fasting, or malnutrition [27]. Like GDF15, FGF21 levels have been shown to increase during obesity and T2D [28], and this paradoxical increase in FGF21 levels in people with obesity suggests a FGF21-resistant state [29]. FGF21 levels also increase with age regardless of the health state [19]. In line with these findings, GDF15 has been proposed as a potential biomarker of biological aging and T2D in humans, featuring GDF15 and FGF21 analogs as potential treatments for obesity and its complications [18, 21, 23, 30].

Therefore, based on the hypothesis that the increased levels of GDF15 and FGF21 associated with obesity and aging suggest a state of resistance to their activity, and that these cytokines may serve as potential predictors of T2D, this study aimed to analyze the association between serum GDF15 and FGF21 concentrations and obesity and T2D in the context of aging. Additionally, it aimed to evaluate their potential as biomarkers for identifying individuals with T2D.

## Materials and methods

### Patient selection and study design

A cohort of 405 White participants (33 with normal weight and NG (NW–NG), 156 with obesity and normoglycemia (OB–NG), 157 with obesity and impaired glucose tolerance (OB–IGT), and 59 with obesity and T2D (OB–T2D)), aged 47 ± 13 year (range 21–70), 55% females, recruited from patients attending to the Obesity Unit and the Department of Endocrinology and Nutrition at the Clínica Universidad de Navarra, were selected to include in a retrospective case–control study to evaluate the serum concentrations of GDF15 and FGF21 and its relationship with obesity, T2D and aging. Participants were newly diagnosed with altered glucose homeostasis either IGT or T2D, following the criteria of the American Diabetes Association [31] and, therefore, were not receiving anti-diabetic treatments at the time of sampling.

The inclusion criteria were 21–70-year-old males and females, body mass index (BMI) between 18.5 and 24.9 kg/m^2^ for NW volunteers and BMI ≥ 30.0 kg/m^2^ for participants with obesity, and absence of psychiatric pathology. The exclusion criteria were severe systemic diseases not related to obesity, such as cancer, infectious diseases, or severe nephropathy, pregnancy or lactation, people who have severe eating disorders, and people whose freedom is under legal or administrative requirement. All participants signed the informed consent to participate in the study. The study was carried out in accordance with the Declaration of Helsinki and was approved by the University of Navarra’s Ethical Committee (2014.129, 2020.041, and 2021.123).

### Anthropometric and body composition measurements

Body weight and composition were measured wearing a swimming suit and cap to the nearest 0.1 kg with a digital scale while the height was determined with a Holtain stadiometer (Holtain Ltd., Crymych, UK) to the nearest 0.1 cm. BMI was calculated by dividing weight in kilograms by the square of height in meters. To measure the waist circumference, a non-elastic tape was placed between the iliac crest and the rib cage. Blood pressure was measured at least three times at the right upper arm after a 5-min rest with the patient in the semi-sitting position with a sphygmomanometer. The data used in the study are the mean of three different measurements. Body density was calculated by air-displacement-plethysmography (Bod-Pod®, COSMED, Rome, Italy), and body fat percentage (BF%) was calculated from body density by means of the Siri equation as previously described [32].

### Serum biochemistry

Serum samples were collected after 8−h of fasting in the morning. Serum biochemistry was analyzed as previously reported [32–35]. Serum glucose, alanine aminotransferase (ALT), aspartate aminotransferase (AST), uric acid, and γ−glutamyltransferase (GGT) were measured by an analyzer (Modular P800, Roche, Basel, Switzerland). Insulin was determined by an enzyme−amplified chemiluminescence assay (Immulite®, Diagnostic Products, Los Angeles, CA, USA). Total cholesterol and triglyceride (TG) levels were measured by enzymatic spectrophotometric methods (Roche). The homeostatic model assessment of insulin resistance (HOMA−IR), the quantitative insulin sensitivity check index (QUICKI), and the triglycerides and glucose index (TyG) were calculated as indirect measures of insulin resistance or insulin sensitivity. The AST/ALT ratio was used as an indicator of hepatic steatosis and fatty liver disease [36].

GDF15 (DGD150, R&D Systems, Minneapolis, MN, USA), FGF21 (RD191108200R, BioVendor, Brno, Czech Republic), leptin (RD191001100, BioVendor), adiponectin (RD191023100, BioVendor), and paraoxonase 1 (PON1) (ELH-PON1, RayBiotech, Norcross, GA, USA) were measured in serum samples by ELISA following the manufacturer’s protocols. The intraassay coefficients of variation were 2.3%, 2.0%, 5.9%, 4.9%, and < 10%, respectively, while the interassay coefficients of variation were 5.4%, 3.3%, 5.6%, 6.7%, and < 12%, respectively. Malondialdehyde (MDA) was estimated by the measurement of thiobarbituric acid reactive substances (TBARS) in serum samples [37].

### Statistical analysis

The program G*Power (edition 3.1.9.4) was used to determine sample size calculation. Data are presented as mean ± SD. The normal distribution of the data was assessed using the Kolmogorov–Smirnov test. Differences between groups were analyzed by one-way ANOVA followed by Fisher’s least significant difference (LSD) post hoc tests, two-way ANOVA, or analysis of covariance (ANCOVA) when adjusting for covariates. Correlations between variables were analyzed by Pearson’s correlation coefficients (*r*)*.* Receiver operating characteristic (ROC) curve analyses were performed to evaluate the predictive value of the variables under study. The mean area under the ROC curves and the best cut-off points were calculated. Statistical analysis was conducted using GraphPad Prism 8.0 software (San Diego, CA, USA) and SPSS version 25 (IBM SPSS Statistics, Chicago, IL, USA). A *P* value lower than 0.05 was considered statistically significant.

## Results

### Clinical characteristics of the population

Clinical features of the participants are shown in Table 1. Anthropometric measurements including body weight, BMI, BF%, waist circumference, hip circumference, waist-to-hip ratio (WHR), and waist-to-height ratio (WHtR) were significantly higher in the groups conformed by individuals with obesity compared to the NW–NG group. Mean values of systolic and diastolic blood pressure were also significantly higher in individuals with obesity. Participants with obesity and T2D showed the highest concentrations of glucose and insulin, along with higher HOMA-IR and lower QUICKI values. Individuals with obesity showed elevated levels of TG and cholesterol, reduced HDL-cholesterol concentrations, and a reduction in the AST/ALT ratio, accompanied by higher GGT levels, suggestive of hepatic steatosis. Lower PON1 levels, an enzyme with antioxidant function, were observed in individuals with obesity, although this did not reach statistical significance compared with the NW–NG group. Finally, MDA concentrations, a marker of oxidative stress, were significantly higher in the OB–NG group compared to the other groups.

**Table 1:** Anthropometric and biochemical characteristics of volunteers

Variable	NW–NG	OB–NG	OB–IGT	OB-T2D	*P*
*n*	33	156	157	59	
Age (years)	37.2 ± 12.1	47.2 ± 14.3***	48.6 ± 12.8***	51.4 ± 10.7***^,†^	** < 0.001**
Body weight (kg)	66.3 ± 7.2	101.9 ± 21.5***	112.0 ± 25.3***^,†††^	110.9 ± 26.5***^,†^	** < 0.001**
Height (m)	1.66 ± 0.07	1.66 ± 0.11	1.67 ± 0.10	1.65 ± 0.10	0.817
BMI (kg/m^2^)	24.1 ± 0.8	36.7 ± 5.9***	40.2 ± 7.2***^,†††^	40.4 ± 7.8***^,††^	** < 0.001**
Body fat (%)	34.1 ± 6.6	46.0 ± 8.4***	48.4 ± 8.2***^,†^	47.6 ± 8***	** < 0.001**
Waist circumference (cm)	81 ± 6	113 ± 14***	122 ± 16***^,†††^	123 ± 17***^,†††^	** < 0.001**
Hip circumference (cm)	101 ± 4	120 ± 11***	124 ± 13***^,††^	123 ± 16***	** < 0.001**
WHR	0.80 ± 0.07	0.95 ± 0.09***	0.98 ± 0.09***^,†††^	1.00 ± 0.08***^,†††^	** < 0.001**
WHtR	0.49 ± 0.03	0.68 ± 0.08***	0.73 ± 0.09***^,†††^	0.74 ± 0.10***^,†††^	** < 0.001**
SBP (mm Hg)	107 ± 14	126 ± 17***	132 ± 18***^,††^	137 ± 21***^,†††^	** < 0.001**
DBP (mm Hg)	67 ± 8	79 ± 10***	82 ± 10***^,††^	85 ± 10***^,†††^	** < 0.001**
Glucose (mg/dL)	89 ± 9	96 ± 11***	101 ± 11***^,†††^	122 ± 24***^,†††,§§§^	** < 0.001**
Glucose 2-h OGTT (mg/dL)	87 ± 16	113 ± 17***	163 ± 18***^,†††^	250 ± 49***^,†††,§§§^	** < 0.001**
Insulin ($\mu$U/mL)	6.4 ± 3.3	12.6 ± 10.1***	17.3 ± 11.3***^,†††^	21.6 ± 16.8***^,†††^	** < 0.001**
Insulin 2-h OGTT ($\mu$U/mL)	53.0 ± 24.5	91.1 ± 56***	147.3 ± 75.8***^,†††^	136.8 ± 77.4***^,†††^	** < 0.001**
HOMA-IR	1.40 ± 0.76	2.99 ± 2.39***	4.37 ± 3.14***^,†††^	6.71 ± 5.73***^,†††,§§^	** < 0.001**
QUICKI	0.38 ± 0.04	0.34 ± 0.04***	0.32 ± 0.04***^,†††^	0.31 ± 0.03***^,†††,§§^	** < 0.001**
TyG index	8.0 ± 0.5	8.5 ± 0.5***	8.7 ± 0.4***^,†††^	9.1 ± 0.5***^,†††,§§§^	** < 0.001**
Triglycerides (mg/dL)	74.3 ± 34.2	117.8 ± 58.0***	132.1 ± 69.4***^,†^	158.5 ± 69.1***^,†††,§^	** < 0.001**
Cholesterol (mg/dL)	185.4 ± 37.2	210.1 ± 43.2**	201.7 ± 42.4*	202.5 ± 36.0*	** < 0.05**
LDL-cholesterol (mg/dL)	101.9 ± 28.6	129.3 ± 36.5***	123.8 ± 37.8**	122.4 ± 34.6**	** < 0.01**
HDL-cholesterol (mg/dL)	68.6 ± 14.3	57.2 ± 16.7***	51.5 ± 14.1***^,††^	48.4 ± 12.8***^,†††^	** < 0.001**
Uric acid (mg/dL)	4.4 ± 1.4	5.7 ± 1.4***	6.0 ± 1.5***^,†^	6.1 ± 1.5***^,†^	** < 0.001**
ALT (IU/L)	13.2 ± 6.9	22.5 ± 13.1***	25.4 ± 13.6***	31.9 ± 19.2***^,†††,§^	** < 0.001**
AST (IU/L)	14.1 ± 5.0	16.6 ± 6.4*	17.6 ± 7.8**	19.8 ± 11.9**	** < 0.01**
AST/ALT ratio	1.20 ± 0.51	0.82 ± 0.24***	0.77 ± 0.25***	0.67 ± 0.19***^,†††,§§^	** < 0.001**
GGT (IU/L)	14.3 ± 13.0	22.6 ± 16.3*	26.7 ± 22.5**	40.7 ± 33.9***^,†††,§§§^	** < 0.001**
PON1 (ng/mL)	210.0 ± 86.0	181.9 ± 76.9	185.6 ± 72.1	178.7 ± 72.0	0.275
MDA (μM/L)	0.93 ± 0.32	1.11 ± 0.38**	1.02 ± 0.41^†^	0.96 ± 0.4^†^	** < 0.05**

Data are mean ± SD. Statistical differences between groups were analyzed by one-way ANOVA followed by LSD *post hoc* tests. Statistically significant differences are highlighted in bold, *NW* normal weight, *NG* normoglycemia, *OB* obesity, *IGT* impaired glucose tolerance *T2D,* type 2 diabetes, *BMI* body mass index, *WHR* waist-to-hip ratio, *WHtR* waist-to-height ratio, *SBP* systolic blood pressure, *DBP* diastolic blood pressure, *HOMA-IR* homeostatic model assessment of insulin resistance, *QUICKI* quantitative insulin sensitivity check index, *TyG index* triglycerides and glucose index, *ALT* alanine aminotransferase, *AST* aspartate aminotransferase, *GGT* γ-glutamyltransferase, *PON1* paraoxonase-1, *MDA* malondialdehyde

*P < 0.05, **P < 0.01, and ***P < 0.001 vs. NW–NG; ^†^*P* < 0.05, ^††^*P* < 0.01, and ^†††^*P* < 0.01 vs. OB-NG; §*P* < 0.05, §§*P* < 0.01, and §§§*P* < 0.001 vs. OB–IGT

### Serum GDF15 and FGF21 concentrations increase in obesity and further increase in obesity-associated T2D

**Fig. 1:**
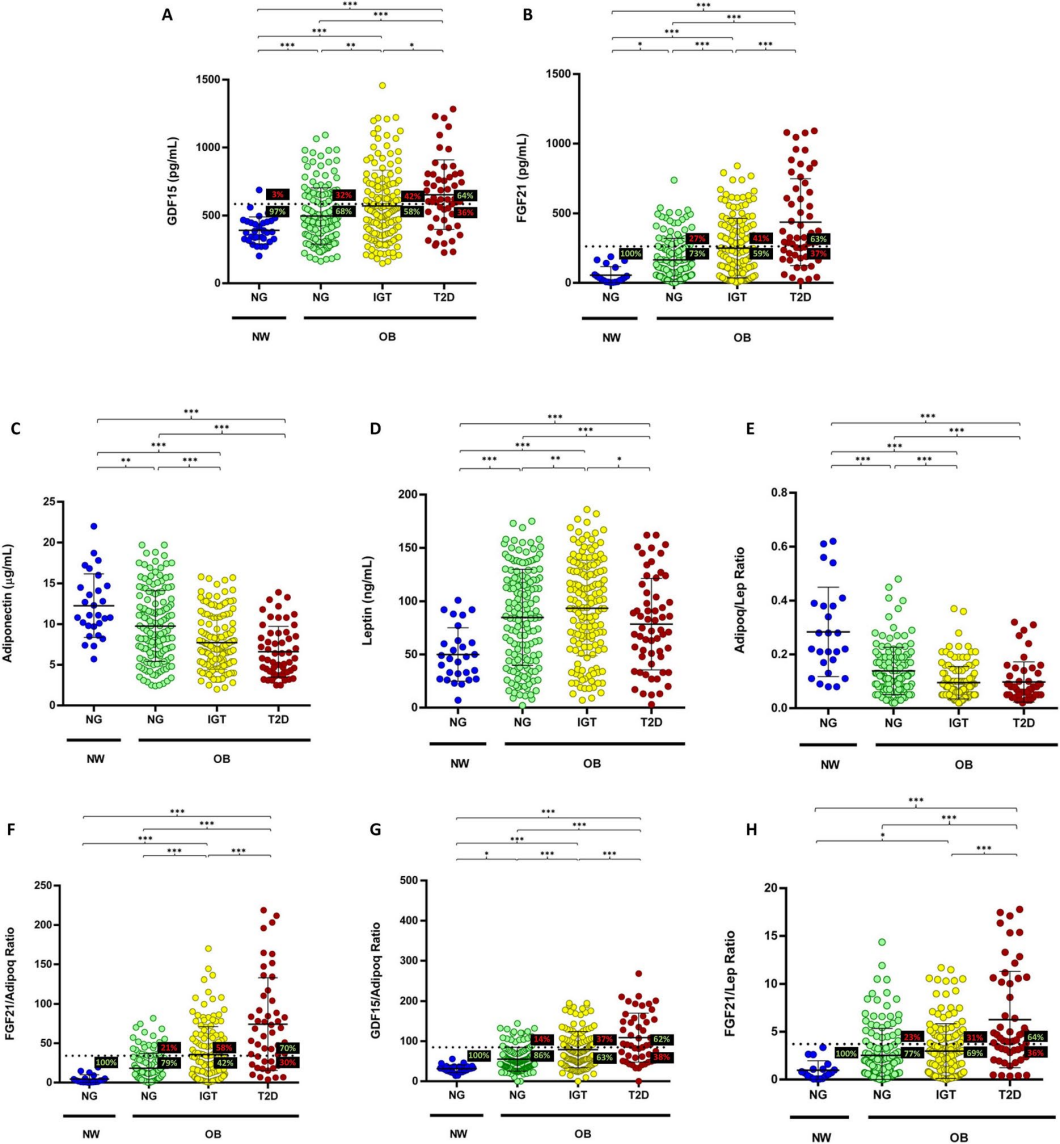
Serum levels and ratios of different metabolic markers in relation to the weight and the glycemic status. Serum levels of **A** GDF15, **B** FGF21, **C** adiponectin, **D** leptin, **E** adiponectin/leptin, **F** FGF21/adiponectin, **G** GDF15/adiponectin, and **H** FGF21/leptin ratios. Horizontal discontinuous lines denote the cut-offs proposed to define T2D for each molecule or ratio, 585.0 pg/mL for GDF15, 262.5 pg/mL for FGF21, 34.07 for FGF21/adiponectin, 84.73 for GDF15/adiponectin, and 3.72 for FGF21/leptin. The percentage of participants above or under the cut-off value is indicated in each group. Bars represent the mean ± SD. Statistical differences between groups were analyzed by one-way ANOVA followed by LSD *post hoc* tests. *P < 0.05, **P < 0.01, and ***P < 0.001. NW, normal weight; OB, obesity; NG, normoglycemia; IGT, impaired glucose tolerance; T2D, type 2 diabetes

Significantly higher circulating concentrations of GDF15 and FGF21 were observed in individuals with obesity and NG compared to the NG–NW group (Fig. 1A, B). Serum levels of both GDF15 and FGF21 were further elevated in participants with IGT and T2D. Due to the important role that leptin and adiponectin play in metabolism, the serum levels of both molecules were measured. Adiponectin levels were progressively lower in individuals with obesity as glucose homeostasis became impaired (Fig. 1C), while leptin concentrations were higher in the OB groups compared to the NW–NG group (Fig. 1D). Consequently, we observed a progressive reduction in the adiponectin/leptin ratio with the impairment of glucose homeostasis (Fig. 1E).

**Fig. 2:**
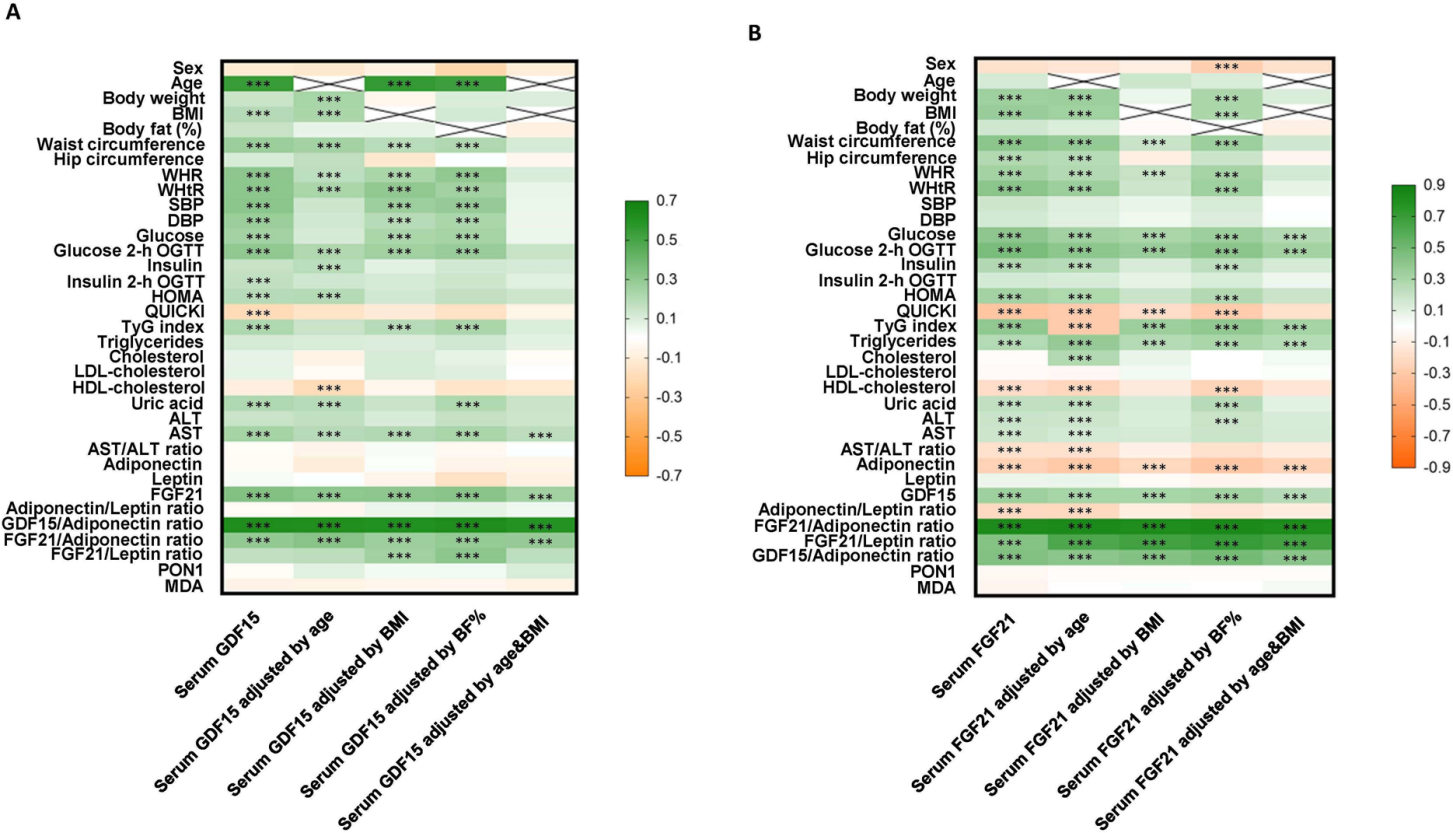
Correlations between GDF15 and FGF21 levels with anthropometric and biochemical variables. Heatmap showing the correlations between **A** serum GDF15 levels and **B** serum FGF21 levels and anthropometric and biochemical variables, before and after adjusting by age, BMI, BF%, or age and BMI. GDF15, growth differentiation factor 15; FGF21, fibroblast growth factor 21; BMI, body mass index; WHR, waist-to-hip ratio; WHtR, waist-to-height ratio; SBP, systolic blood pressure; DBP, diastolic blood pressure; HOMA-IR, homeostatic model assessment of insulin resistance; QUICKI, quantitative insulin sensitivity check index; TyG index, TG and glucose index; ALT, alanine aminotransferase; AST, aspartate aminotransferase; PON1, paraoxonase-1; MDA, malondialdehyde. The color scale represents the value of the Pearson’s correlation coefficients. Green color indicates positive correlations while orange color indicates negative correlations. For correlation with gender, male = 1 and female = 2 were used. The *P* value ***P < 0.001 is emphasized with asterisks

Figure 2 summarizes the associations of GDF15 and FGF21 levels with different variables before and after adjusting by age, BMI, BF%, or age and BMI. A strong positive correlation between GDF15 and FGF21 (*r* = 0.34, *P* < 0.001) was observed, which remained significant after adjustments. Additionally, both cytokines showed a significant positive correlation with age. FGF21 maintained more significant correlations than GDF15 with variables associated with insulin resistance, such as glucose, insulin, HOMA-IR, and QUICKI, and showed significant positive correlations with circulating TG and with the TyG index (Supplementary Tables 1 and 2). Moreover, a significant negative correlation between FGF21 and adiponectin (*r* =  − 0.20, *P* < 0.001) and between FGF21 and the adiponectin/leptin ratio (*r* =  − 0.24, *P* < 0.001) was observed.

### Serum GDF15 and FGF21 levels show positive correlation with glucose and aging

**Fig. 3:**
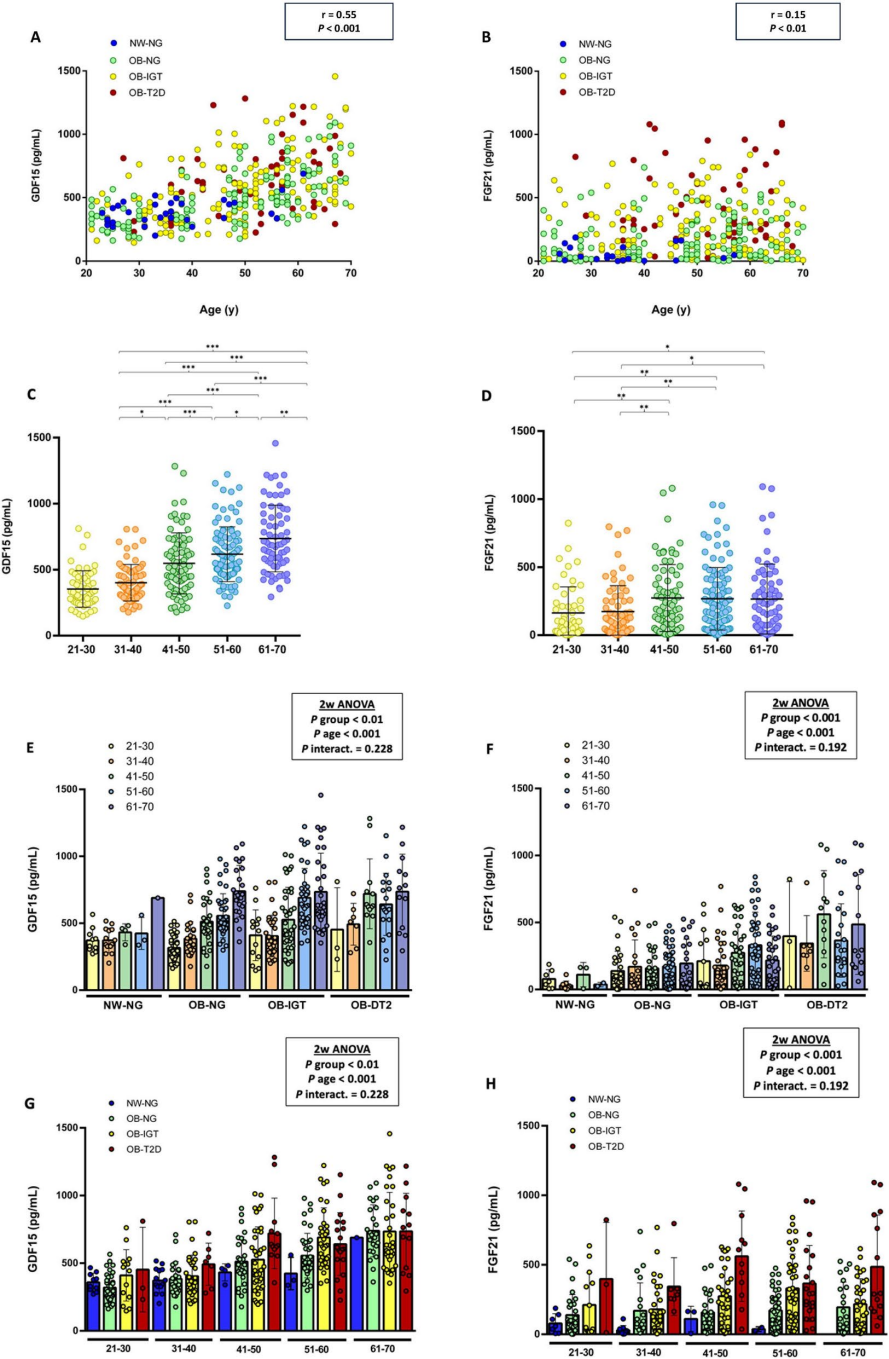
Analysis of the correlations between serum GDF15 and FGF21 levels with age, weight, and glycemic status. Scatter diagrams showing the correlation between serum levels of **A** GDF15 and **B** FGF21 levels with age. Circulating concentrations of **C** GDF15 and **D** FGF21 in participants classified by age. Serum levels of **E** GDF15 and **F** FGF21 in each glycemic group depending on age. Serum levels of **G** GDF15 and **H** FGF21 in each age group depending on the weight and the glycemic status. Pearson’s correlation coefficient (*r*) and *P* value or main effects by two-way ANOVA are indicated. Bars represent the mean ± SD. Statistical differences between groups were analyzed by two-way ANOVA or one-way ANOVA followed by LSD *post hoc* tests. *P < 0.05, **P < 0.01, and ***P < 0.001

Since GDF15 and FGF21 have been described as potential biomarkers of aging, we decided to further study the association between both cytokines and age in the context of different ponderal and glycemic statuses. As previously described [19, 20], both cytokines showed a positive correlation with age (Figures 2 and 3A, B). However, while serum GDF15 levels were progressively higher with aging (Fig. 3C), serum FGF21 levels reached their highest values in the 41–50 years age group (Fig. 3D). In the groups shaped by individuals with obesity, serum GDF15 levels were higher in older age groups, regardless of the glycemic status (Fig. 3E). Nevertheless, the mean serum GDF15 levels in each age group was higher in the OB–IGT and OB–T2D groups than in the OB–NG group. The youngest age groups (21–40 years) exhibited smaller differences in serum GDF15 concentrations based on glycemic status compared to the oldest age groups (41–70 years) (Fig. 3E). Within the oldest age groups, the impact of glycemic status was less evident than the impact of the obesity status. The oldest group (61–70 years) showed similar values of serum GDF15 levels, and these values were higher than those in the other age groups (Fig. 3G). Unlike GDF15, serum FGF21 concentrations did not show this clear trend with aging (Fig. 3F); however, they exhibited notably higher levels in each age group depending on glycemic status (Fig. 3H).

**Fig. 4:**
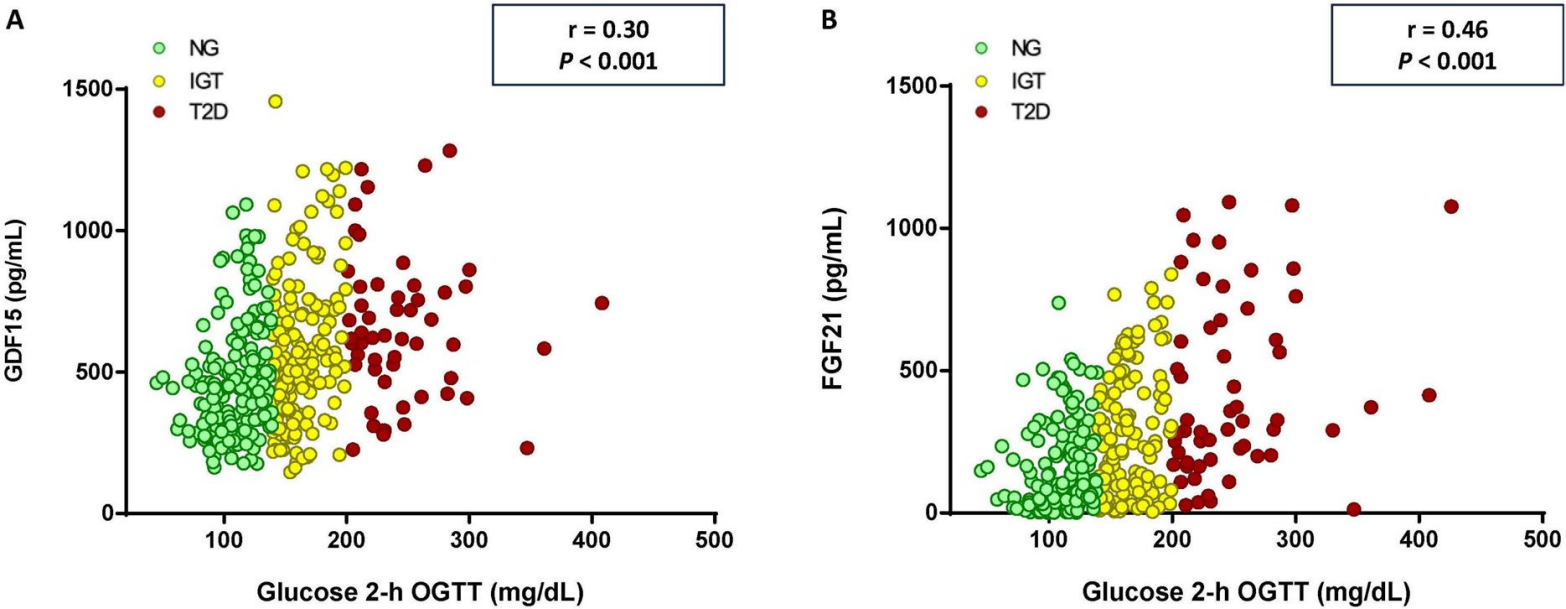
Scatter diagrams showing the correlation between glucose 2 h after the oral glucose tolerance test (OGTT) and circulating concentrations of **A** GDF15 and **B** FGF21

Regarding the differences between GDF15 and FGF21 in relation to the glycemic status, we studied the correlation between both cytokines and the glucose levels 2 h after OGTT. Both cytokines showed significant positive correlations with 2-h glucose levels FGF21 (*r* = 0.46, *P* < 0.001; GDF15 *r* = 0.30, *P* < 0.001) (Fig. 4). Importantly, circulating concentrations of both cytokines were influenced by sex, with higher levels found in males than in females for GDF15 (570.7 ± 253.4 pg/mL for males, 513.6 ± 227.4 pg/mL for females; *P* < 0.05) and for FGF21 (278.3 ± 240.8 pg/mL for males, 199.4 ± 215.0 pg/mL for females; *P* < 0.01) (Supplementary Fig. 1).

To evaluate whether the observed associations of GDF15 and FGF21 with age were independent of body composition, we conducted ANCOVA analyses including body weight and BF% as covariates. Body weight showed a significant independent effect for both GDF15 and FGF21 (*P* < 0.001), whereas BF% was not significant for GDF15 (*P* = 0.292) but was significant for FGF21 (*P* < 0.01). In a combined model including body weight and BF% as covariates, age and body weight remained significant predictors for both GDF15 and FGF21 (*P* < 0.001), while BF% was not significant (*P* = 0.426 for GDF15, *P* = 0.517 for FGF21). These findings indicate that, although body weight significantly influences circulating levels of both GDF15 and FGF21, the positive association of these biomarkers with age remains independent of body composition. This supports the presence of aging-related regulatory mechanisms affecting their concentrations beyond changes in weight and BF%. To further explore the factors influencing GDF15 and FGF21 levels, we conducted ANCOVA analyses including metabolic group and age as factors. Age had a significant independent effect on GDF15 levels (*P* < 0.001), but not on FGF21 (*P* = 0.209). In contrast, metabolic group was a significant predictor for both GDF15 and FGF21 (*P* < 0.01 and *P* < 0.001, respectively). These findings suggest that, while GDF15 is strongly associated with age independent of metabolic status, FGF21 levels are primarily influenced by metabolic status rather than age.

### FGF21/adiponectin ratio as a potential biomarker of type 2 diabetes

**Fig. 5:**
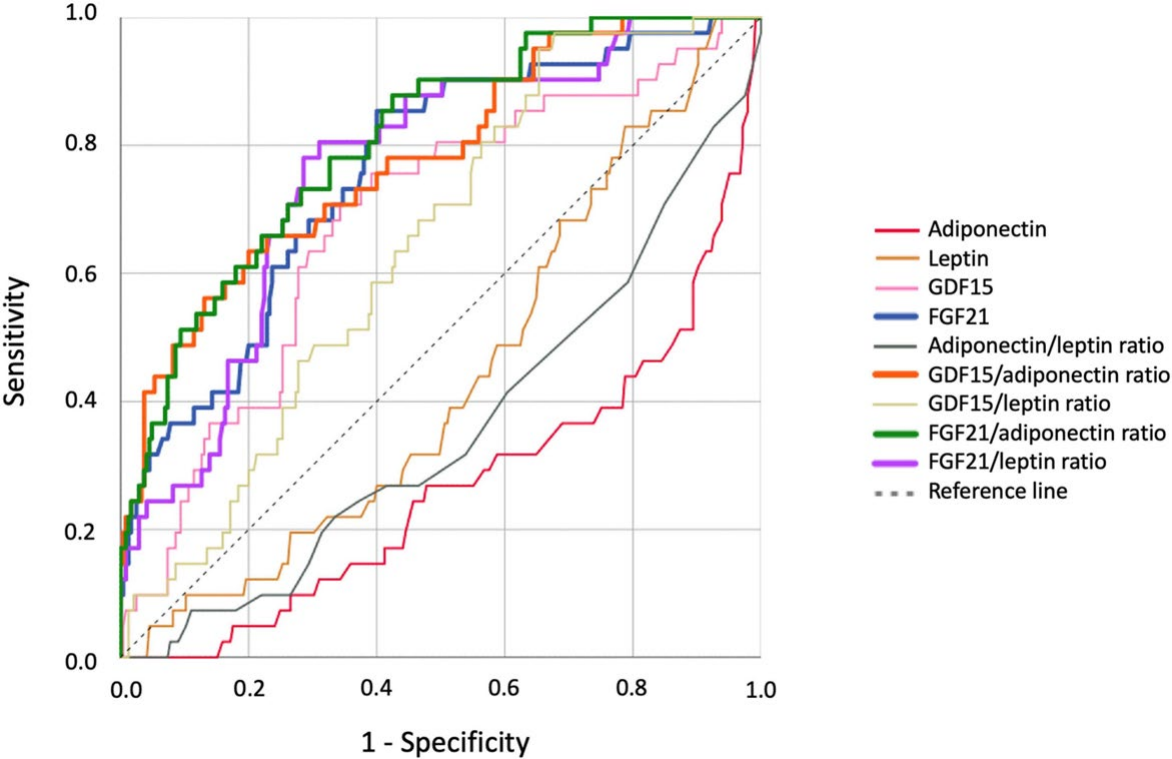
Receiver operating characteristic (ROC) curves for identifying T2D using different serum biomarkers and their ratios. ROC curves of adiponectin, leptin, GDF15, and FGF21, and of adiponectin/leptin, GDF15/adiponectin, GDF15/leptin, FGF21/leptin, and FGF21/adiponectin ratios, to detect T2D

In order to identify the most effective biomarker for detecting T2D in our cohort, we performed ROC analysis with adiponectin, leptin, GDF15, FGF21, adiponectin/leptin ratio, GDF15/adiponectin ratio, GDF15/leptin ratio, FGF21/adiponectin ratio, and FGF21/leptin ratio. The area under the curve (AUC) was 0.268, 0.438, 0.683, 0.760, 0.359, 0.779, 0.642, 0.807, and 0.762, respectively (Fig. 5, Supplementary Table 3). The highest AUC was 0.807 for the FGF21/adiponectin ratio, with a Youden index of 0.429 based on the highest sensitivity and specificity values together, which were 71% and 72%, respectively. The Youden index is a summary measure of the effectiveness of a diagnostic test, calculated as sensitivity + specificity − 1. It identifies the optimal cut-off point by maximizing the balance between sensitivity and specificity [38].

High AUC values were also observed for the GDF15/adiponectin (AUC = 0.779) and the FGF21/leptin (AUC = 0.762) ratios with a Youden index of 0.430 and 0.406, respectively. The cut-off values associated with the GDF15, FGF21, FGF21/adiponectin, GDF15/adiponectin, and FGF21/leptin Youden indexes were 585.0 pg/mL, 262.5 pg/mL, 34.07, 84.73, and 3.72, respectively (Fig. 1A, , F–H). In practical terms, when we applied the cut-off point of 34.07 for the FGF21/adiponectin ratio in our cohort, we correctly classified 100% of individuals in the NW–NG group, and 79%, 42% and 70% of participants with obesity and NG, IGT, or T2D, respectively. In contrast, T2D is misdiagnosed in 21% and 58% of individuals with obesity and NG or IGT, respectively, and we have 30% false negatives in the OB–T2D group (Fig. 1F). Due to the known differences between sexes in the variables analyzed, separate ROC curves were also performed for women and men, with a similar result to that of the overall cohort analysis (Supplementary Fig. 2).

## Discussion

The burgeoning evidence of the important role that GDF15 and FGF21 play in metabolic alterations has envisioned these cytokines as promising biomarkers for obesity diagnosis and as potential therapeutic targets for treating some obesity-associated complications [39–44]. Hence, we mainly focused on the study of GDF15 and FGF21 in relation to obesity and T2D in the context of aging.

According to previous studies [28, 45], our results showed a positive correlation between GDF15 and FGF21 levels and BMI, as well as with glucose and insulin levels, highlighting their relationship with impaired glycemic control and insulin resistance, both of which are associated with the development of T2D. In relation to the association of GDF15 levels with faster T2D progression [46], we observed that GDF15 levels were higher in people with obesity and T2D than in those with obesity but without T2D. A similar trend was observed for FGF21, with the differences being even more pronounced. This suggests that FGF21 levels are more closely associated with glycemic impairment than GDF15 concentrations.

A positive association between circulating concentrations of GDF15 and FGF21 with age was observed. This is in agreement with the previously reported role of both cytokines as aging biomarkers [47, 48] showing a positive correlation between GDF15 and FGF21 concentrations and age in healthy individuals [19, 20, 49]. We observed that serum GDF15 levels were higher with age in all the groups with obesity, independently of the glycemic status, although the difference was more pronounced in those with worse glycemic control. Serum GDF15 levels were similar in the 61–70 age groups, which could represent the possible resistant state to GDF15 associated with aging. FGF21 reached its highest concentrations in the age group between 41 and 50 years. In this line, we observed that although both cytokines are related to the glycemic status and aging, FGF21 has a stronger association with glycemic status while GDF15 has a stronger relation with aging. Although the correlations are stronger, a physiological relevance of this difference in association cannot be concluded. Nonetheless, our results support the observation that FGF21 levels seem to be more closely associated with metabolic impairment whereas GDF15 concentrations appear to be more strongly associated with aging. Our data support a state of GDF15 and FGF21 resistance in obesity, T2D, and aging.

To further study the association between GDF15 or FGF21 and T2D, given that both cytokine levels are elevated in different disorders, which makes it difficult to use them alone to predict the onset of a specific disease [10, 13, 27, 50, 51], additional variables were included to identify more accurately the presence of T2D. Given the relationship between AT dysfunction, obesity, and T2D, we decided to include in our study the adiponectin/leptin ratio, previously identified as a potential biomarker of AT dysfunction [34, 52] and insulin resistance in individuals with T2D [53]. In fact, an essential role of both leptin and adiponectin has been described in metabolic syndrome. The main function of adiponectin is to improve insulin sensitivity while leptin primarily regulates food intake and metabolism [54]. Moreover, while leptin is upregulated in people with obesity, adiponectin is downregulated [54, 55]. In this sense, we found a significant negative correlation between FGF21 and adiponectin and the adiponectin/leptin ratio. Considering their important role in metabolic homeostasis, we included the FGF21/leptin and FGF21/adiponectin ratios in the analysis, along with the GDF15/leptin and GDF15/adiponectin ratios, as potential T2D biomarkers. To study which variable could be the best detector of T2D, ROC analyses were performed. Among all the variables, the FGF21/adiponectin ratio had the highest AUC, closely followed by the GDF15/adiponectin and the FGF21/leptin ratios, all of which showed higher diagnostic accuracy than FGF21, GDF15, adiponectin, or leptin alone. The diagnostic performance of GDF15 alone was lower compared to the one described by Roy D et al. in their study population [56]. This is probably because many participants of their study were on treatment, mostly on metformin, which increases GDF15 levels to induce weight loss [57]. The ability of FGF21 to identify the presence of T2D is consistent with the statistically significant correlation observed between FGF21 and glucose.

Albeit to our knowledge, this is the first study of the FGF21/adiponectin ratio in the White population; this ratio was previously proposed as a good indicator of T2D in the Chinese population [58]. The GDF15/adiponectin ratio [59] was also proposed as a potential biomarker for metabolic syndrome in Han Chinese people [60]. Herein, our data support the role of both ratios in other populations and determine a close association between high FGF21/adiponectin and GDF15/adiponectin ratios and the presence of T2D. While well-established biomarkers are currently used for the detection of T2D, our findings suggest that the FGF21/adiponectin and GDF15/adiponectin ratios may provide additional value as early indicators of metabolic stress, potentially identifying individuals at risk of T2D before the clinical onset of the disease. Although this cross-sectional study cannot establish their predictive value for future diabetes onset, it provides a necessary foundation for future longitudinal research. Prospective studies analyzing the association between these ratios and the development of T2D may clarify their potential use as biomarkers for the development of T2D in people with obesity, helping to prevent the onset of T2D earlier than with current tools.

Our study has some potential limitations. Firstly, all the participants were White people, and to study whether our conclusions could be extended to other populations would be necessary. Secondly, although we recruited a large cohort, age stratification led to a small sample size in some experimental groups. Thirdly, the absence of an independent validation cohort limits our ability to confirm the predictive value of GDF15 and FGF21 for future age-related disease risk. Despite these limitations, the large sample size of our cohort and the well-characterized population allow for robust conclusions to be obtained in our study.

Taken together, our findings manifest a marked relationship between GDF15 and FGF21 levels with T2D, together with an association with aging. The elevated levels of both cytokines in these conditions could imply a resistance mechanism to the effects of both GDF15 and FGF21, possibly due to reduced sensitivity to them. However, this increase could also be mediated by lower clearance or by a compensatory mechanism in response to the changes caused by obesity, T2D, and aging. Interestingly, circulating GDF15 concentrations are more associated with aging while FGF21 levels are more related to the metabolic status. Finally, we propose FGF21/adiponectin ratio as a better marker of T2D than FGF21 or GDF15 levels alone.

## Supplementary Information

Below is the link to the electronic supplementary material.

ESM 1 (PDF 729 KB)

## Data Availability

The datasets used and/or analyzed during the current study are available from the corresponding author on reasonable request.
