# Supplementary material for: Different association of GDF15 and FGF21 with glycemic status and aging in the context of obesity
Source: GeroScience. 2025 Aug 19;48(2):2931–44. doi: 10.1007/s11357-025-01830-3 (PMC12972332; doi:10.1007/s11357-025-01830-3)
Supplement: Supplementary file 1 — (PDF 729 KB) [file 11357_2025_1830_MOESM1_ESM.pdf]

Geroscience

Different association of GDF15 and FGF21 with glycemic status and aging in the context of obesity

Laura Salmón-Gómez · Victoria Catalán · Beatriz Ramírez · Maite Aguas-Ayesa · Amaia Rodríguez · Sara Becerril · Jorge Baixauli · Sonsoles Gutierrez-Medina · Carmen Mugueta · Inmaculada Colina · Carolina M. Perdomo · Camilo Silva · Javier Escalada · Gema Frühbeck · Javier Gómez-Ambrosi

Corresponding author:
Javier Gómez-Ambrosi, PhD
Metabolic Research Laboratory
Clínica Universidad de Navarra
Irunlarrea 1
31008 Pamplona
Spain
Phone: +34 948 425600 (ext. 806567)
e-mail: jagomez@unav.es

Supplementary Table 1. Analysis of the correlation between GDF15 and anthropometric and biochemical variables, unadjusted and after adjusting by age, BMI, BF% or age and BMI.

	Serum GDF15		Serum GDF15 adjusted by age		Serum GDF15 adjusted by BMI		Serum GDF15 adjusted by BF%		Serum GDF15 adjusted by age&BMI	
**Variable**	**r**	***P* value**	**r**	***P* value**	**r**	***P* value**	**r**	***P* value**	***r***	***P* value**
**Sex**	-0.118	**<0.05**	-0.136	**<0.05**	-0.080	0.147	-0.224	**<0.001**	-0.105	0.058
**Age**	0.545	**<0.001**	-	-	0.552	**<0.001**	0.527	**<0.001**	-	-
**Body weight**	0.149	**<0.01**	0.241	**<0.001**	-0.048	0.385	0.102	0.065	0.095	0.088
**BMI**	0.200	**<0.001**	0.226	**<0.001**	-	-	0.120	**<0.05**	-	-
**Body fat (%)**	0.155	**<0.01**	0.063	0.253	0.064	0.250	-	-	-0.084	0.127
**Waist circumference**	0.274	**<0.001**	0.250	**<0.001**	0.192	**<0.001**	0.216	**<0.001**	0.112	**<0.05**
**Hip circumference**	0.108	**<0.05**	0.175	**<0.01**	-0.132	**<0.05**	0.003	0.961	-0.045	0.419
**WHR**	0.310	**<0.001**	0.181	**<0.001**	0.229	**<0.001**	0.298	**<0.001**	0.102	0.065
**WHtR**	0.312	**<0.001**	0.231	**<0.001**	0.301	**<0.001**	0.255	**<0.001**	0.060	0.280
**SBP**	0.318	**<0.001**	0.133	**<0.05**	0.267	**<0.001**	0.287	**<0.001**	0.054	0.329
**DBP**	0.272	**<0.001**	0.126	**<0.05**	0.195	**<0.001**	0.227	**<0.001**	0.050	0.366
**Glucose**	0.249	**<0.001**	0.111	**<0.05**	0.224	**<0.001**	0.247	**<0.001**	0.053	0.340
**Glucose 2-h OGTT**	0.303	**<0.001**	0.216	**<0.001**	0.256	**<0.001**	0.279	**<0.001**	0.152	**<0.01**
**Insulin**	0.159	**<0.01**	0.187	**<0.001**	0.078	0.161	0.123	**<0.05**	0.118	**<0.05**
**Insulin 2-h OGTT (μU/mL)**	0.168	**<0.001**	0.128	**<0.05**	0.115	**<0.05**	0.137	**<0.05**	0.086	0.121
**HOMA**	0.193	**<0.001**	0.203	**<0.001**	0.123	**<0.05**	0.166	**<0.01**	0.136	**<0.05**
**QUICKI**	-0.200	**<0.001**	-0.150	**<0.01**	-0.111	**<0.05**	-0.164	**<0.01**	-0.065	0.244
**TyG index**	0.218	**<0.001**	0.150	**<0.01**	0.193	**<0.001**	0.227	**<0.001**	0.097	0.081
**Triglycerides**	0.114	**<0.05**	0.102	0.065	0.095	0.087	0.125	**<0.05**	0.076	0.172
**Cholesterol**	0.062	0.223	-0.067	0.224	0.109	**<0.05**	0.067	0.227	-0.024	0.670
**LDL-cholesterol**	0.071	0.164	-0.028	0.608	0.109	**<0.05**	0.090	0.103	-0.002	0.973
**HDL-cholesterol**	-0.096	0.060	-0.194	**<0.001**	-0.053	0.342	-0.140	**<0.05**	-0.126	**<0.05**
**Uric acid**	0.217	**<0.001**	0.194	**<0.001**	0.144	**<0.01**	0.219	**<0.001**	0.144	**<0.01**
**ALT**	0.150	**<0.01**	0.168	**<0.01**	0.106	0.056	0.160	**<0.01**	0.138	**<0.05**
**AST**	0.243	**<0.001**	0.193	**<0.001**	0.210	**<0.001**	0.235	**<0.001**	0.185	**<0.001**
**AST/ALT ratio**	-0.023	0.646	-0.059	0.288	0.023	0.677	-0.042	0.445	0.003	0.960
**GGT**	0.197	**<0.001**	0.171	**<0.01**	0.185	**<0.001**	0.218	**<0.001**	0.151	**<0.01**
**Adiponectin**	-0.030	0.571	-0.126	**<0.05**	0.012	0.824	-0.052	0.347	-0.085	0.123
**Leptin**	0.027	0.601	-0.010	0.859	-0.064	0.247	-0.154	**<0.01**	-0.094	0.089
**FGF21**	0.340	**<0.001**	0.317	**<0.001**	0.301	**<0.001**	0.323	**<0.001**	0.254	**<0.001**
**Adipoq/Lep ratio**	-0.036	0.504	-0.010	0.871	0.056	0.346	0.098	0.097	0.081	0.171
**GDF15/Adipoq ratio**	0.625	**<0.001**	0.631	**<0.001**	0.619	**<0.001**	0.645	**<0.001**	0.613	**<0.001**
**FGF21/Adipoq ratio**	0.280	**<0.001**	0.308	**<0.001**	0.252	**<0.001**	0.276	**<0.001**	0.262	**<0.001**
**FGF21/Lep ratio**	0.310	**<0.001**	0.251	**<0.001**	0.269	**<0.001**	0.333	**<0.001**	0.224	**<0.001**
**PON1**	-0.024	0.646	0.082	0.138	0.035	0.523	0.034	0.536	0.103	0.062
**MDA**	-0.067	0.197	-0.066	0.230	-0.053	0.343	-0.052	0.349	-0.074	0.184

GDF15, growth differentiation factor 15; BMI, body mass index; BF%, body fat percentage WHR, waist-to-hip ratio; WHtR, waist-to-height ratio; SBP, systolic blood pressure; DBP, diastolic blood pressure; HOMA, homeostatic model assessment; QUICKI, quantitative insulin sensitivity check index; TyG index, triglycerides, and glucose index; ALT, alanine aminotransferase; AST, aspartate aminotransferase; GGT, gamma-glutamyl transferase; Adipoq, adiponectin; Lep, leptinPON1, paraoxonase-1; MDA, malondialdehyde. Data are Pearson's correlation coefficients and associated *P* values. For correlation with gender, male = 1 and female =2 was used. Significant correlations are highlighted in bold.

Supplementary Table 2. Analysis of the correlation between FGF21 and anthropometric and biochemical variables, unadjusted and after adjusting by age, BMI, BF% or age and BMI.

	**Serum FGF21**		**Serum FGF21 adjusted by age**		**Serum FGF21 adjusted by BMI**		**Serum FGF21 adjusted by BF%**		**Serum FGF21 adjusted by age&BMI**	
**Variable**	**r**	***P* value**	**r**	***P* value**	**r**	***P* value**	**r**	***P* value**	***r***	***P* value**
**Sex**	-0.171	**<0.01**	-0.151	**<0.01**	-0.099	0.080	-0.272	**<0.001**	-0.177	**<0.05**
**Age**	0.146	**<0.01**	-	-	0.177	**<0.01**	0.130	**<0.05**	-	-
**Body weight**	0.336	**<0.001**	0.342	**<0.001**	0.068	0.232	0.286	**<0.001**	0.128	**<0.05**
**BMI**	0.358	**<0.001**	0.332	**<0.001**	-	-	0.289	**<0.001**	-	-
**Body fat (%)**	0.167	**<0.01**	0.126	**<0.05**	-0.031	0.589	-	-	-0.090	0.117
**Waist circumference**	0.410	**<0.001**	0.368	**<0.001**	0.194	**<0.001**	0.343	**<0.001**	0.163	**<0.01**
**Hip circumference**	0.272	**<0.001**	0.255	**<0.001**	-0.094	0.096	0.181	**<0.01**	-0.058	0.317
**WHR**	0.318	**<0.001**	0.268	**<0.001**	0.204	**<0.001**	0.317	**<0.001**	0.155	**<0.01**
**WHtR**	0.409	**<0.001**	0.345	**<0.001**	0.176	**<0.01**	0.340	**<0.001**	0.084	0.143
**SBP**	0.171	**<0.01**	0.119	**<0.05**	0.074	0.190	0.139	**<0.05**	0.003	0.957
**DBP**	0.153	**<0.01**	0.107	0.060	0.056	0.326	0.123	**<0.05**	0.004	0.939
**Glucose**	0.398	**<0.001**	0.328	**<0.001**	0.303	**<0.001**	0.343	**<0.001**	0.266	**<0.001**
**Glucose 2-h OGTT**	0.463	**<0.001**	0.401	**<0.001**	0.356	**<0.001**	0.405	**<0.001**	0.318	**<0.001**
**Insulin**	0.271	**<0.001**	0.254	**<0.001**	0.141	**<0.05**	0.229	**<0.001**	0.148	**<0.01**
**Insulin 2-h OGTT (μU/mL)**	0.153	**<0.01**	0.137	**<0.05**	0.088	0.120	0.133	**<0.05**	0.058	0.313
**HOMA**	0.320	**<0.001**	0.283	**<0.001**	0.184	**<0.01**	0.266	**<0.001**	0.186	**<0.01**
**QUICKI**	-0.332	**<0.001**	-0.295	**<0.001**	-0.197	**<0.001**	-0.291	**<0.001**	-0.174	**<0.01**
**TyG index**	0.386	**<0.001**	-0.295	**<0.001**	0.343	**<0.001**	0.387	**<0.001**	0.317	**<0.001**
**Triglycerides**	0.263	**<0.001**	0.369	**<0.001**	0.260	**<0.001**	0.290	**<0.001**	0.255	**<0.001**
**Cholesterol**	-0.019	0.715	0.274	**<0.001**	0.076	0.178	-0.005	0.930	0.042	<0.470
**LDL-cholesterol**	-0.033	0.528	-0.047	0.404	0.043	0.452	-0.007	0.904	0.012	0.830
**HDL-cholesterol**	-0.199	**<0.001**	-0.237	**<0.001**	-0.125	**<0.05**	-0.248	**<0.001**	-0.146	**<0.05**
**Uric acid**	0.218	**<0.001**	0.219	**<0.001**	0.141	**<0.01**	0.249	**<0.001**	0.094	0.101
**ALT**	0.184	**<0.001**	0.173	**<0.01**	0.128	**<0.05**	0.194	**<0.001**	0.140	**<0.05**
**AST**	0.178	**<0.001**	0.154	**<0.01**	0.151	**<0.01**	0.183	**<0.01**	0.148	**<0.01**
**AST/ALT ratio**	-0.174	**<0.001**	-0.172	**<0.01**	-0.086	0.131	-0.177	**<0.01**	-0.111	0.053
**GGT**	0.244	**<0.001**	0.243	**<0.001**	0.232	**<0.001**	0.280	**<0.001**	0.218	**<0.001**
**Adiponectin**	-0.261	**<0.001**	-0.287	**<0.001**	-0.203	**<0.001**	-0.306	**<0.001**	-0.235	**<0.001**
**Leptin**	0.066	0.212	0.069	0.212	-0.062	0.258	-0.097	0.079	-0.068	0.219
**GDF15**	0.340	**<0.001**	0.317	**<0.001**	0.301	**<0.001**	0.323	**<0.001**	0.254	**<0.001**
**Adipoq/Lep ratio**	-0.238	**<0.001**	-0.244	**<0.001**	-0.114	0.055	-0.176	**<0.01**	-0.111	0.060
**FGF21/Adipoq ratio**	0.854	**<0.001**	0.857	**<0.001**	0.837	**<0.001**	0.856	**<0.001**	0.841	**<0.001**
**FGF21/Lep ratio**	0.721	**<0.001**	0.763	**<0.001**	0.771	**<0.001**	0.831	**<0.001**	0.765	**<0.001**
**GDF15/Adipoq ratio**	0.432	**<0.001**	0.369	**<0.001**	0.341	**<0.001**	0.398	**<0.001**	0.316	**<0.001**
**PON1**	-0.043	0.426	-0.019	0.738	-0.021	0.715	-0.027	0.641	-0.020	0.731
**MDA**	-0.064	0.232	0.003	0.961	0.010	0.857	0.001	0.981	0.037	0.521

FGF21, fibroblast growth factor 21; BMI, body mass index; BF%, body fat percentage WHR, waist-to-hip ratio; WHtR, waist-to-height ratio; SBP, systolic blood pressure; DBP, diastolic blood pressure; HOMA, homeostatic model assessment; QUICKI, quantitative insulin sensitivity check index; TyG index, triglycerides, and glucose index; ALT, alanine aminotransferase; AST, aspartate aminotransferase; GGT, gamma-glutamyl transferase; GDF15, growth differentiation factor 15; Adipoq, adiponectin; Lep, leptin; PON1, paraoxonase-1; MDA, malondialdehyde. Data are Pearson's correlation coefficients and associated *P* values. For correlation with gender, male = 1 and female =2 was used. Significant correlations are highlighted in bold.

Supplementary Table 3. Area under the curve (AUC) obtained in the receiver operating characteristic (ROC) analysis to detect the presence of type 2 diabetes.

Variable	AUC	*P*
Adiponectin	0.268	<0.001
Leptin	0.438	0.204
GDF15	0.683	<0.001
FGF21	0.760	<0.001
Adiponectin/leptin ratio	0.359	<0.01
GDF15/adiponectin ratio	0.779	<0.001
GDF15/leptin ratio	0.642	<0.01
FGF21/adiponectin ratio	0.807	<0.001
FGF21/leptin ratio	0.762	<0.001

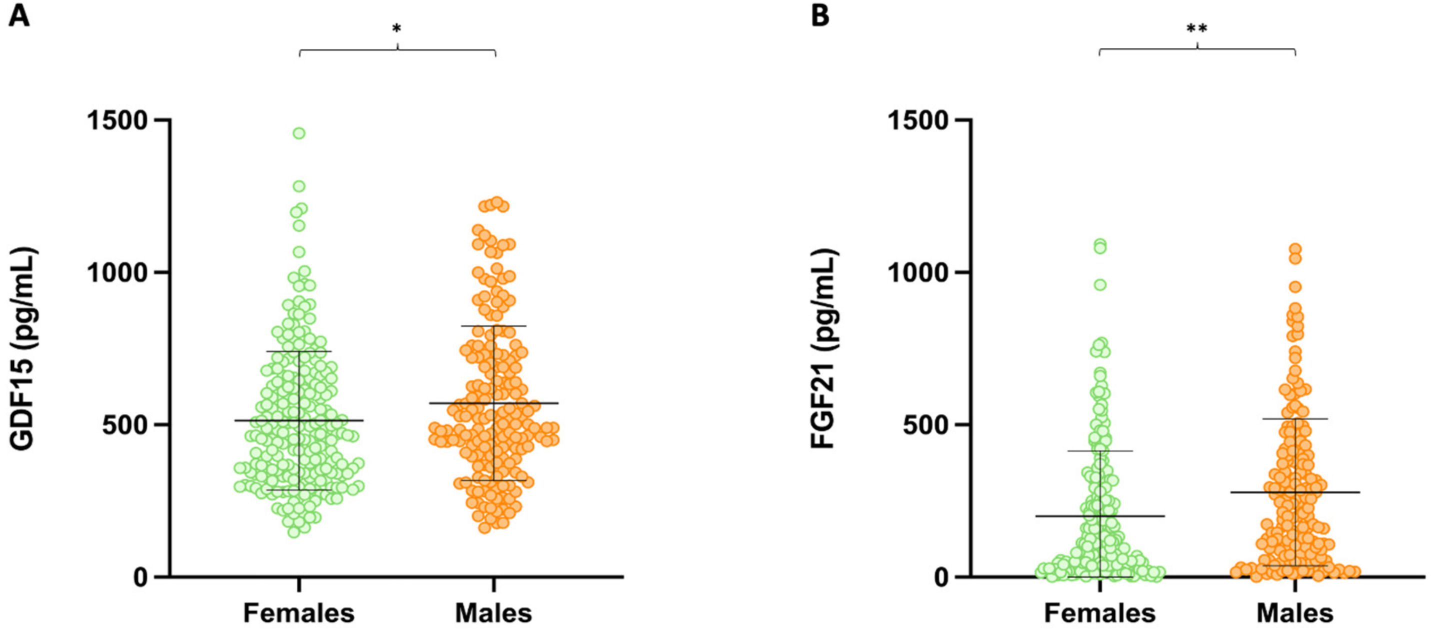

Supplementary Figure 1. Serum levels of (A) GDF15 and (B) FGF21 depending on sex. Statistical differences between groups were analyzed by unpaired Student's *t* tests. *$P<0.05$ and **$P<0.01$.

A

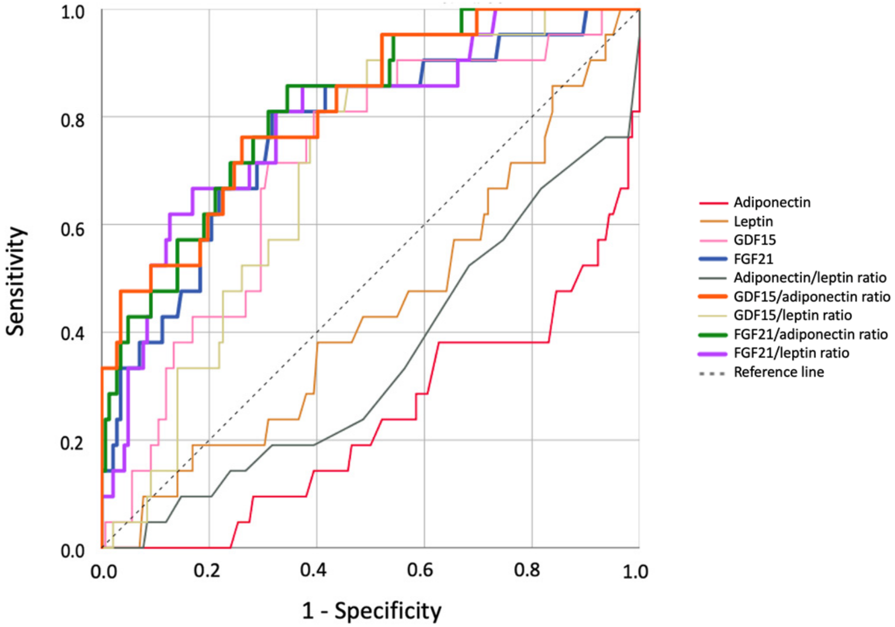

Variable	AUC	*P*
Adiponectin	0.237	<0.001
Leptin	0.441	0.385
GDF15	0.705	<0.01
FGF21	0.772	<0.001
Adiponectin/leptin ratio	0.348	<0.05
GDF15/adiponectin ratio	0.815	<0.001
GDF15/leptin ratio	0.706	<0.01
FGF21/adiponectin ratio	0.817	<0.001
FGF21/leptin ratio	0.793	<0.001

B

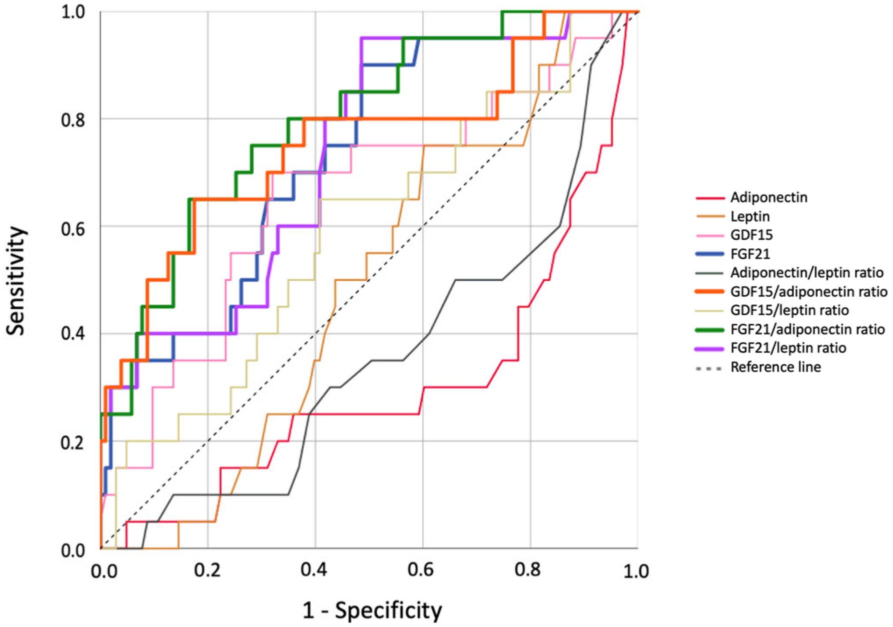

Variable	AUC	*P*
Adiponectin	0.296	<0.01
Leptin	0.491	0.896
GDF15	0.656	<0.05
FGF21	0.734	<0.001
Adiponectin/leptin ratio	0.356	<0.05
GDF15/adiponectin ratio	0.754	<0.001
GDF15/leptin ratio	0.589	0.210
FGF21/adiponectin ratio	0.797	<0.001
FGF21/leptin ratio	0.735	<0.001

Supplementary Figure 2. Receiver operating characteristic (ROC) curves of Adiponectin, Leptin, GDF15 and FGF21, and of Adiponectin/Leptin, GDF15/Adiponectin, GDF15/Leptin, FGF21/Leptin and FGF21/Adiponectin ratios to predict T2D with their AUC and *P* values in (A) women and (B) men.
